# When support is needed: Social support solicitation and provision in an online alcohol use disorder forum

**DOI:** 10.1177/2055207617704274

**Published:** 2017-05-22

**Authors:** Yan Liu, Rachel Kornfield, Bret R Shaw, Dhavan V Shah, Fiona McTavish, David H Gustafson

**Affiliations:** 1 Department of Life Sciences Communication, University of Wisconsin-Madison, Madison, Wisconsin, USA; 2 School of Journalism & Mass Communication, University of Wisconsin-Madison, Madison, Wisconsin, USA; 3 Center for Health Enhancement System Studies, University of Wisconsin-Madison, Madison, Wisconsin, USA

**Keywords:** Alcohol use disorder, alcoholism, emotional expression, online support groups, self-disclosure, social support

## Abstract

**Background:**

Obtaining adequate social support presents a challenge for many in addiction recovery. Increasingly, individuals in recovery use online forums to exchange support with peers, yet it is unclear which help-seeking strategies most effectively recruit peer support, and which forms of support are most valued by recipients.

**Methods:**

This study applied quantitative content analysis to examine social support solicitation and delivery in an online forum for alcohol use disorder (AUD). We compared the frequency with which peers provided informational, emotional, and companionship support after solicitations that: (1) were direct or indirect, (2) disclosed positive or negative emotions, and (3) mentioned or did not mention recovery problems. We assessed likelihood that recipients would express gratitude after receiving each type of support, and assessed whether the “match” between solicitation and disclosure styles influenced rates of gratitude expression.

**Results:**

Emotional disclosures, whether positive or negative, received the highest volume of supportive replies. Emotional support was the most common response to solicitations overall, and was disproportionately offered after recipients disclosed positive emotions. Informational support was disproportionately offered after recipients disclosed negative emotions or recovery problems, or explicitly requested help. Regardless of their solicitation style, recipients expressed more gratitude after receiving emotional support than other support types.

**Conclusions:**

Providing emotional support was common in an online AUD forum, and precipitated expressing gratitude from recipients to support providers. The results may be helpful in guiding participants to more effectively obtain and provide recovery support in online forums.

## Introduction

Alcohol use disorder (AUD) represents an important public health issue. Nearly 88,000 people died from excessive alcohol use annually from 2006 to 2010, making alcohol-related death the third leading preventable cause of death in the United States.^1^ In a recent survey, 10% of all American adults aged 18 and older considered themselves to be in recovery from an alcohol or drug abuse problem.^2^ Negative consequences of alcohol abuse are not restricted to deteriorating health and mortality, but also include the substantial distress incurred among those with AUD and their loved ones,^1,3^ and a massive economic burden, with costs of alcohol abuse in the United States exceeding $249 billion in 2010.^1^

Individuals fare best in overcoming substance abuse problems when they have others in their lives to provide social support. Social support involves a range of behaviors that aid individuals in adjusting to and coping with challenges, including providing information and advice, expressing validation and caring, or making oneself available as a companion. In the context of alcohol abuse, social support has been associated with various benefits, including recipients’ increased self-efficacy, decreased depression and stress, and reduced drinking.^4,5^ However, as alcohol abuse can involve social stigma and relationship dysfunction,^6^ the necessary level and quality of social support may not be readily available.

Through advances in technology, individuals with substance abuse problems may now expand their social networks by going online, including through websites and mobile applications that facilitate communication among peers who share substance abuse problems.^7^ In a health context, peer-to-peer communication has potential benefits including mutual understanding and bonding based on a shared identity, provision of relevant guidance and information, and reciprocity wherein individuals act as both providers and receivers of support.^8,9^ While peer-to-peer support represents a longstanding facet of substance abuse recovery, it has historically operated primarily in face-to-face environments, such as meetings of Alcoholics Anonymous and other in-person mutual help groups; only limited research has thus far assessed how peer-to-peer recovery support operates in internet-supported, text-based forums. Furthermore, research on social support, both offline and online, has focused on benefits of receiving different forms of support or expressing empathy to others, neglecting the role played by styles of support solicitation.^10,11^

The present study examines how supportive replies are matched to solicitation styles in an online AUD forum. Specifically, we examine the amount of informational, emotional, and companionship support received after direct solicitations of help and indirect solicitations through self-disclosure. We further assess the likelihood that recipients will express gratitude after each form of support, allowing us to gauge the expressed value of received support. This study advances our understanding of online social support by stipulating the conditions under which social support flows toward and benefits its recipients in a real-world recovery context. Moreover, it has implications for the design of online forums, including developing guidance for individuals seeking and providing recovery support.

## Literature review

Individuals with chronic illnesses increasingly utilize computer-mediated communication environments for health support.^12^ Nearly 20% of American internet users used online groups for health-related peer support in 2012.^13^ Peer support forums offer numerous benefits, including their potential for anonymity, convenient access across time and space, and connections between participants with common circumstances.^14-16^ Since online venues typically involve less social risk than face-to-face ones, they provide suitable environments within which to discuss sensitive or stigmatized issues.^17,18^ Through their asynchronous and text-based format, online forums also grant participants necessary tools to carefully plan and edit their messages, which can encourage thoughtful exchanges.^19^ Given these considerations, some have argued that online environments are well suited to fostering prosocial and supportive exchanges (see e.g. Walther et al.^20^; Tanis^21^).

Emerging evidence suggests that peer support forums provide a number of benefits to their participants, especially those facing serious health concerns or lacking social support and acceptance in their offline lives.^22^ For instance, spending more time in online support groups has been associated with enhanced access to informational and emotional support among those with HIV/AIDS.^23^ Results of panel studies have shown that women with breast cancer who participated in an online support group had positive psychological change over 6 months,^24^ and a randomized controlled trial of an internet-based breast cancer intervention including peer-to-peer discussion found increased social support and information-seeking competence after 5 months.^25^ A recent qualitative meta-synthesis study showed that online health communities enhanced empowerment in coping with disease and allowed participants to expand their access to social ties in order to exchange information and other support that would be unavailable offline.^26^

Despite these promising findings, other evidence suggests that the effectiveness of peer support varies in online contexts.^27^ Some recent studies suggest that receiving a higher number of online supportive messages through online support groups has minimal beneficial effects on participants’ emotional and physical health outcomes, including in contexts of cancer^10^ and mental health.^28,29^ A number of factors may contribute to these findings. For instance, support effectiveness may require responding to the needs of individual help-seekers, but this may be difficult to accomplish in online settings with anonymous, personally unknown participants. That is, members of online forums typically lack personal relationships with one another (e.g. friend, family) and may therefore not know one another’s personal history or demographic characteristics. In addition, the text-based format of online communication removes nonverbal cues (e.g. facial expression, voice and body language) that provide valuable information when interacting face to face. Especially in this context, obtaining effective support could depend on effectively soliciting it. For instance, individuals in online support groups may reveal more intimate information about themselves in order to compensate for the limited personalizing information available, thus allowing for initiation and maintenance of online relationships.^30,31^ Scholars have proposed that online environments may be suboptimal for support exchange if supporters deploy mundane advice or miscalibrated help.^32^ There is a need, therefore, to identify patterns of online support that recipients find most useful, and to establish how individuals can most effectively solicit support.

Prior research identifies a number of strategies that individuals use to accrue social support. First, directly asking for help or advice can solicit support.^32^ Numerous online forums feature question-asking as one frequently used form of support solicitation, with questions typically beginning new threads.^32,33^ In addition, research has found that messages including lengthy or detailed stories can also be an effective way to solicit replies.^34^ These self-focused stories can demonstrate commonalities between members of a group, which may motivate responses. Moreover, the “triage model” of social support suggests that individuals who convey that they have higher levels of need tend to receive more social support from others over time.^27^

### Matching between self-disclosure and social support

While there are multiple ways to obtain support online, whether support is ultimately helpful may depend on how well it addresses solicitors’ particular concerns or needs. One dominant perspective in the social support literature holds that the appropriateness of support must be assessed within its interactional context. That is, the benefit derived from support depends on the “match” between the type of support one needs and the type one receives, a proposition known as “optimal matching theory”.^35^ Occasionally, individuals make their support needs relatively clear, such as when they ask questions that illuminate informational needs. In other cases, matching support to recipients’ needs relies on information gleaned from the solicitors’ self-disclosure. Jourard and Lasakow define self-disclosure as the “process of making the self known to other persons” (p.91)^36^ By highlighting one’s particular concerns, self-disclosure provides details to guide others in responding in a person-centered manner.^37,38^ Self-disclosure may indirectly convey a need for a variety of support types (e.g. advice, compassion).^39-41^ For instance, individuals report that, by sharing negative emotions, they seek to receive emotional support that provides validation and comfort.^42^

Some evidence supports the position that recipients prefer consistency between their solicitation style and supporters’ replies, as far as the affective or informational focus. Cutrona and her colleagues^43^ conducted an experiment that found that when participants made an emotional disclosure to their spouse, they felt more satisfied when they received emotional support in response. One explanation could be that when individuals disclose their emotions, emotional support provides needed validation and empathy, whereas advice may create distance between receivers and providers.^43^ In another study, Vlahovic and her colleagues^44^ examined patterns of seeking and providing support in an online breast cancer group, finding that when women asked questions, they were more satisfied after receiving informational support relative to emotional support. After emotional disclosures, however, they were equally satisfied with both types of support. Both of these studies suggest that consistency between the solicitation and response can be important, although one study favors matching the emotional dimension of support solicitations to an emotional support reply, whereas the other favors matching the informational dimension of a solicitation to an informational reply.

Complicating this picture, a recent study revealed “mismatched” responses can also bring benefits. Batenburg and Das^45^ conducted an experiment testing the importance of matching supportive responses to an assigned disclosure style. They proposed that individuals assigned to write about their emotions would benefit more from emotional support, whereas individuals assigned to cognitive reappraisal writing (wherein they reconsider the meaning of an event), would benefit from cognitive reappraisal support in which they are encouraged to consider new perspectives. To the contrary, their results showed that those who disclosed their emotions benefited after receiving cognitive reappraisal support but not after receiving emotional support or no response.^45^ They speculated that individuals who disclosed their emotions became immersed in negative affect and failed to generate new perspectives that could aid them in reevaluating their stressors. Cognitive reappraisal support may have helped individuals think about stressors in new ways, relieving distress. Thus, the extant literature is inconclusive with regard to the optimal matching of support to solicitations. Further research is needed to establish whether recipients in online groups favor certain support types, and whether the perceived value of support is moderated by that support’s “match” to their solicitation style.

### Social support in online addiction groups

Recovery involves challenges that make multiple types of support potentially relevant. First, it may involve substantial distress, uncertainty, and stigma that create a need for social integration and validation; these needs are well addressed by emotional support.^46^ In addition, those with alcohol use problems may require informational support to guide decision-making about complex day-to-day recovery issues such as treatment, coping with cravings and emotions, relationships, or legal matters. Informational support may include both referrals to resources (e.g. websites, books, face-to-face meetings) as well as recommendations about a course of action. Finally, those in recovery may seek to extend their relationships beyond the discussion forum. Companionship support, wherein individuals discuss availability to make contact beyond the discussion forum (e.g. phone, text, in person), may contribute to a sense of social belonging.^47^ Regular social contact has been a feature of offline social support groups, including the ongoing and close relationships with “sponsors” in Alcoholics Anonymous. Companionship support could also improve availability of on-demand contact in times of need, such as seeking more instrumental support (e.g. a ride to a medical appointment).^48^

A few recent studies have examined how social support exchange operates in online AUD forums, with results showing evidence of a number of uses, including exchange of the support types above. That is, forums are used for sharing personal experiences, setting shared goals, building empathic relationships, and offering and receiving companionship and information.^49-52^ Some studies have further compared the frequency at which forms of social support were offered, finding that emotional support characterizes a minority of exchanges on public peer-to-peer forums, with informational support being overrepresented.^49^ However, prior studies have evaluated overall frequency of support types, but not the emergence of support within interactions. Therefore, it remains unclear to what extent social support providers are responsive to particular ways their recipients solicit support.

Furthermore, it is largely unknown what effects each type of support may have. We are aware of one study that examined this issue in an online recovery forum, with findings suggesting that receiving emotional support more effectively sustained recipients’ commitment to the online group.^53^ Specifically, individuals who received emotional support were 16% more likely to remain in the support group, whereas those who received informational support were 10% more likely to leave. These findings suggest benefits of emotional support for sustaining online supportive relationships. However, continued participation is an imperfect proxy for support satisfaction since leaving the group could also signal that individuals have satisfied their support needs.

In the present study, we test a more proximal outcome of support receipt: gratitude expression. Not only does gratitude represent a direct acknowledgment of satisfaction with support, but expression of gratitude has also been associated with improvements in social relationships and well-being.^54^ In addition, gratitude is associated with increases in patience and impulse control,^55^ effects that may be relevant to recovery. In the context of AUD, gratitude expression may also help individuals to build their confidence to sustain a clean and sober life.^56^ In short, gratitude expression could generate both inter and intrapersonal conditions that may operate, over time, to reinforce recovery. Indeed, some mobile interventions now focus directly on prompting gratitude expression as a means to enhance well-being.^57^ In this paper, we examine how expressions of gratitude emerge within exchanges where support seekers use a variety of strategies to communicate their support needs.

## Hypotheses

Below, we pose a series of hypotheses about the amount and type of support secured by solicitations, and the likelihood of gratitude expression.

According to the social support “triage model,” support providers use their support offerings strategically, to resolve the most serious problems.^27^ Consistent with this model, we expect to see greater responsiveness to issues conveyed as more serious by solicitations. Direct requests for support make a need for help explicit. Furthermore, given the forum’s designation as a space to support recovery and prevent relapse, we expect that describing recovery problems will also warrant high levels of response. Therefore, we pose the following hypotheses:

H1a: Direct forms of solicitation will receive a greater volume of supportive replies and will have a lower non-response rate, compared with non-direct forms of solicitations (i.e. self-disclosure only).

H1b: Descriptions of recovery problems will receive a greater volume of supportive replies and will have a lower non-response rate, compared with disclosure without recovery problems.

Our expectations about support volume after emotional self-disclosures are less clear due to conflicting evidence. Some work shows support flowing to the most distressed.^27^ However, in other studies, greater disclosure of negative emotion was not associated with peer responsiveness.^34^ Some work shows that posting negative emotions publicly may receive fewer responses when viewed as inappropriate in light of the publicness of a forum,^58^ or when readers simply do not know how to respond, or find it awkward or unpleasant.^59,60^ Whereas the evidence is mixed on supportive responses to negative emotions, we are not aware of work examining the social support obtained by positively valenced, and mixed valence expressions. Therefore, the following research question is posed:

RQ1: How many supportive replies will be received after self-disclosures with negative valence, positive valence, both, and neither?

Next, we examine how the *type* of support received varies according to the style of solicitation. Prior work shows that support seekers’ needs and preferences can be inferred from various cues in dyadic conversations, leading providers to offer different types of support in response.^39,61^ For instance, some have argued that asking questions reveals a desire for informational resources.^11,63^ In our study, direct requests for support, including asking questions or explicitly stating a desire for support, may signal to support providers that solicitors want them to deploy specific information and expertise (i.e. informational support), as opposed to more general responses such as emotional support or companionship. Consistent with the prior literature, the following hypothesis is posed:

H2: Direct support requests will be more likely to receive informational support.

Whereas direct support requests may call for informational support, self-disclosures could be viewed as calling for emotional or informational responses, depending on the context. First, to our knowledge, no published work has explored how peers help each other after self-disclosures about recovery problems. Second, the literature is also unclear on the role of emotional valence in prompting different types of support, since much of the prior work on emotional disclosure has not distinguished between negatively and positively valenced disclosures. Third, companionship support has been relatively less studied compared with emotional support and informational support, as it is less prevalent in health-related online support groups.^22^ Therefore, we ask:

RQ2: Which type of support will be offered after disclosures of recovery problems?

RQ3: Which type of support will be offered after negative or positively valenced emotional disclosures?

RQ4: What is the amount of companionship support received by different solicitation strategies?

Finally, we examine the effects of receiving matched and unmatched support. In this study, support satisfaction is operationalized as sending gratitude to the support providers, a proximal indicator that a support receiver feels satisfied with the support. As the prior work is inconclusive with regard to the effect of “matching” social support to a receiver’s solicitation style, the final set of research questions examine whether gratitude expression varies depending on types of support received, as well as on “match” between solicitation and support provision.

RQ5a: Which support type will receive the highest rate of gratitude?

RQ5b: Does gratitude expression depend on the match between solicitation type and support type?

## Methods

The messages examined in this study were from an online peer-to-peer support forum, representing one component within a mobile health intervention for relapse prevention that was disseminated to a cohort of 170 participants upon completing residential treatment for AUD. Results from a clinical trial of the intervention are reported elsewhere.^65^ The support forum allowed members of the study to start new threads, and to read and reply to others’ messages. Among 170 participants, 130 individuals posted at least one message to the discussion forum over the course of 12 months.

### Coding procedure

Coders were two graduate students and one undergraduate student from a large Midwestern university. These three coders divided between them the 2590 messages that were posted by participants on the peer-to-peer forum during a 12-month study period, and applied the codebook detailed below. The inter-coder reliability was deemed acceptable high, with an average Cohen’s Kappa of 0.78 across all the codes we applied.

### Coding system

#### Support solicitation coding

Among messages beginning a new thread, the coders identified each instance of support solicitation, and designated all applicable subtypes of support solicitation: direct support solicitation and/or self-disclosures.

Direct requests for support involved participants mentioning their need for help from their peers (e.g. “I could really use some help.”) or asking a non-rhetorical question (e.g. “I’m worried I might relapse, what do I do?”).

Self-disclosures involved conveying any personally revealing information, including one’s emotions, opinions/perspectives, or facts about oneself. For messages that were coded as emotional self-disclosure, message valence was further coded; that is, we indicated whether these messages expressed positive emotions, negative emotions, or both. An example of a negatively valenced self-disclosure is: “[…] Sometimes I feel like I'm going crazy. Just needed to vent.” An example of a positively valenced self-disclosure is: “[…] I'm feeling really good and thankful for everyday sober. It's really awesome to see the world through sober eyes. […]” An example of both negatively and positively valenced self-disclosure is: “[…] I feel good about it I have been doing everything I was told to. So I think it will be ok. But still I'm worried.”

In self-disclosure messages, references to specific recovery problems were also noted. In such messages, the writer describes a current or past threat to well-being and/or recovery efforts. The comment may express feeling vulnerable in general, or may outline a specific problem (e.g. “Ugh, I have been fighting with my partner and really feeling discouraged lately. not sure I can do this at all”).

#### Social support coding

Within threads that began with a solicitation, all responses were coded for whether they offered social support and, if so, the type(s) of support offered: informational, emotional, or companionship. The coding system was adapted from one developed by Cohen and Wills (1985) that considers instrumental or tangible, cognitive (which we call “informational”), emotional, and social companionship support.^62^ Tangible assistance does not suit online groups,^63^ and was omitted from our study.

#### Emotional support

Emotional support refers to messages fostering feelings of comfort and leading the recipient to believe that he or she is understood, respected, or loved. The commenter may convey that he/she feels empathy for the recipient, or may offer well wishes or prayers and encouragement. Given the online context, we further specified that affectionate emoticons (e.g. smiley faces), nicknames (e.g. sweetheart), and affectionate slang (e.g. xoxo) may represent emotional support. Examples include: “I feel for you. Sounds so hard, so I will be praying for you!” and “Hi, and welcome to the team!”

#### Informational support

Informational support refers to information, knowledge, or advice to help the recipient “understand his or her world and to adjust to changes within it.”^64^ The supporter may convey how he or she thinks it would be useful or appropriate to think about or respond to a given situation, or may suggest other resources including websites, features within the mobile intervention, or recovery events or services. Examples from this study are: “I strongly suggest you call your sponsor before you start your day” and “Getting into the big book this week … pg. 83-89 is pretty amazing stuff if you let it be.”

#### Companionship support

Companionship support involves the support provider describing availability to provide contact beyond the discussion board, defined by any mention of meeting in person or conversing by phone, private message, or using any other communication channel beyond the study forum. Examples are: “you can call or text me anytime.” “Come to [my town] to a meeting. Call me and I will meet u.”

For messages that fell into multiple coding categories (e.g. a supportive message might include both emotional support and informational support), all the relevant codes were applied.

#### Gratitude

Gratitude occurs when the support solicitor expresses thanks to others for assistance and/or support they have offered in the thread. Only gratitude expressions occurring after a supportive message and specifically naming the support provider were included (e.g. “Thank you for the wise words, Elmo53!”).

### Analysis plan

To identify the relationships between support solicitations and their responses, the intended recipients of social support messages were designated according to a simple algorithm. Specifically, for threads that began with a self-disclosure or direct support request, all replies that offered social support were coded as being directed to the individual who started the thread. Manual review by a human coder of 100 social support replies confirmed that this approach accurately captured the recipients of supportive messages (k = .92).

Thus, for each support solicitation, we were able to calculate (1) the number of replies received, and (2) the proportion of replies that fell in each of our social support categories. Independent samples *T*-tests were used to compare the number of responses received for direct and indirect support, and for disclosures of recovery problems versus other disclosures. Analysis of variance (ANOVA) was used to compare the number of responses received according to self-disclosure valence, defined as (1) only negative emotional disclosure (2) only positive emotional disclosure (3) both and (4) neither. In addition, Chi-square tests were used to compare the proportion of support responses of each type that followed different solicitation styles. Finally, to examine predictors of gratitude expression, logistic regressions were conducted to test (1) the association between each type of support and the expression of gratitude and (2) the interaction between types of social support solicitation and types of support received.

## Results

### User profile

Of the 130 participants, 56% of them were male, and 80% were white or Caucasian. The mean age of these participants was 38 years (SD = 9.72), with a range of 20 to 64 years. As far as education, 5.6% never attended high school; 54.7% had a high school diploma, GED, or some high school; 27.0% had completed some college, and 11.9% had a college degree or higher.

These 130 participants posted a total of 2590 messages over the course of a year, with 849 messages that started new threads. Of these, 778 (92%) met our criteria of being support solicitations, with most of the rest offering general unsolicited social support to the community. Of the 778 support solicitations, 119 were direct support solicitations, and 764 messages included some form of self-disclosure. Thus, the vast majority of direct solicitations also included self-disclosures. Of total self-disclosures, 465 included emotional self-disclosure and 593 included a recovery problem. Within emotional disclosure messages, 241 disclosure messages had only positive valence, 174 disclosure messages had only negative valence and 50 disclosure messages had mixed valence.

In total, support solicitations messages received 1340 social support replies (mean = 1.72 per solicitation). Of these replies, 756 included informational support, 1000 included emotional support, and 121 included companionship support. Thus, emotional support was the most common type of support offered. Some 162 support solicitation messages (16%) received no social support responses from peers, though forum moderators may have responded.

### Number of support messages received after solicitations for support

There was a statistically significant difference in the number of replies generated by direct (mean = 2.08) and indirect (mean = 1.66) support solicitations, *t* (776) = 2.862, *p* = 0.004 (Table 1). We also found a statistically significant difference in replies generated by discussion of recovery problems (mean = 2.15) versus other self-disclosures (mean = 1.49), *t* (762) = 6.039, *p* < 0.001. Finally, according to our ANOVA results, there were significantly different response rates according to self-disclosure valence, *F* (3, 760) = 7.458, *p* < 0.001) (Table 2). According to LSD post-hoc tests, disclosures with only negative valence received significantly more replies on average (2.09) than disclosures that were only positive (1.79) or that did not mention emotions (1.45) (*p* = 0.043 and <0.001, respectively). In addition, positively valenced disclosures received more replies than disclosures that did not mention emotions (*p* = 0.007). Support solicitations that had mixed emotional valence were not significantly different from the other groups in the number of replies.

**Table 1.** Response rate of direct and indirect solicitation, disclosure with/out recovery problem.

	Response				
Solicitation	*n*	*M*	*SD*	*t*	*df*
Direct	119	2.076	1.730	2.862**	776
Indirect	659	1.659	1.409		
Self-disclosure with recovery problem	276	2.149	1.569	6.039***	762
Self-disclosure without recovery problem	488	1.492	1.368		

*Note.* *p < 0.05, **p < 0.01, ***p ≤ 0.001

**Table 2.** Response rate of emotional disclosure with different valences.

	Response			
Emotional disclosure	*n*	*M*	*SD*	*F*
Only positive	241	1.793_a_	1.428	7.458***
Only negative	174	2.086_b_	1.531	
Both	50	1.860	1.784	
Neither	299	1.448_c_	1.378	

Note. *p < 0.05, **p < 0.01, ***p ≤ 0.001 Means with differing subscripts within columns are significantly different at the *p* < 0.05 based on Fisher’s LSD post-hoc paired comparisons.

We also used Chi-square tests of proportion to compare the fraction of solicitations that did not receive any response. Direct support solicitations were less likely to receive a non-response (14%) than indirect solicitations (22%), but this difference did not achieve statistical significance (*p* = 0.065). Non-response was less common after recovery problem disclosures (13%) than after disclosures not mentioning a recovery problem (25%) (*p* < 0.001). Non-response was more common after comments with non-emotional disclosures (27%) and mixed emotional disclosures (22%) than after positive (17%) and negative disclosures (15%) (*p* = 0.012).

### Matching

**Figure 1.**
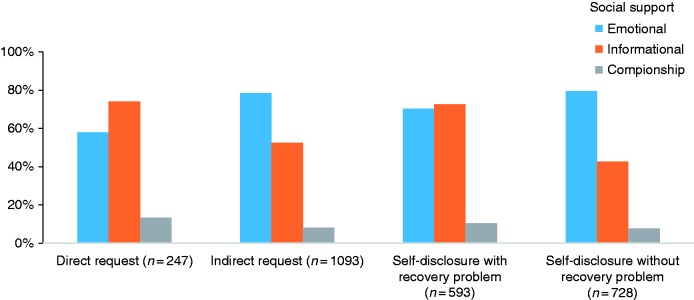
Support types between direct and indirect solicitations; between disclosures with and without recovery problems.

**Figure 2.**
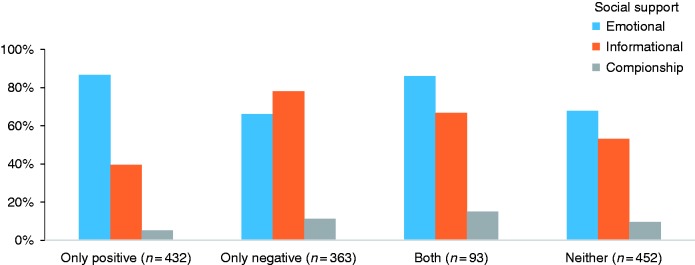
Support types among emotional disclosures with different valences.

Compared with direct requests (57.9% of replies), indirect request messages received a higher proportion of emotional support (78.4% of replies) (Table 3). Messages wherein individuals disclosed recovery problems received a higher proportion of informational support (72.5% of replies), compared with disclosures without recovery problems (42.7% of replies), and received a lower proportion of emotional support (70.3% of replies) relative to disclosures without recovery problems (79.5%) (Table 4; Figure 1). With regard to valence, emotional support responses were more common following positive-only disclosures (86.6% of replies) (Table 4; Figure 2). They were less common following disclosure of negative-only emotions (66.1% of replies). Messages that only disclosed negative emotions were more likely to receive informational support (78.0% of replies) than other emotional messages. Companionship support was more common following messages that disclosed mixed negative and positive emotions (15.1% of replies) (Table 5).

**Figure 3.**
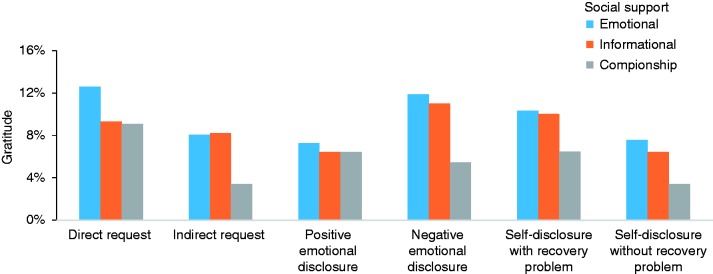
Gratitude expression among emotional disclosures with different valences; between direct and indirect solicitations; between disclosures with and without recovery problems.

**Table 3.** Support received after direct and indirect solicitation.

		Solicitation		
Social support		Direct support request	Indirect support request (self-disclosure only)	χ2
Emotional	*n*	143	857	
	%	57.89	78.41	44.773***
Informational	*n*	183	573	
	%	74.08	52.42	38.458***
Companionship	*n*	33	88	
	%	13.36	8.05	6.913**

Note. *p < 0.05, **p < 0.01, ***p ≤ 0.001

**Table 4.** Support received after disclosures with/out revealing recovery problem.

		Self-disclosure		
Social support		Self- disclosure with recovery problem	Self- disclosure without recovery problem	χ2
Emotional	*n*	417	579	
	%	70.32	79.53	43.862***
Informational	*n*	430	311	
	%	72.51	42.71	121.952***
Companionship	*n*	62	57	
	%	10.45	7.82	2.795

Note. *p < 0.05, **p < 0.01, ***p ≤ 0.001

**Table 5.** Support received after emotional disclosures with different valences.

	Emotional disclosure					
Social support		Only positive	Only negative	Both	Neither	χ2
Emotional	*n*	374a	240_b_	80_a_	306_b_	64.285***
	%	86.57	66.12	86.02	67.7	
Informational	*n*	171_a_	283_b_	62_b,c_	240_c_	
	%	39.58	77.96	66.67	53.1	124.312***
Companionship	*n*	23_a_	41_b_	14_b_	43_a,b_	
	%	5.32	11.29	15.05	9.51	13.726**

Note. *p < 0.05, **p < 0.01, ***p ≤ 0.001 Differing subscripts within rows are significantly different at the *p* < 0.05.

### Gratitude

A logistic regression was built to assess which types of support were associated with gratitude expression (Table 6; Figure 3). Model 1 showed that emotional support was a significant predictor of gratitude expression (OR = 1.87, *p* < 0.05), whereas other types of support did not show significant associations with expressing gratitude. Emotional social support was still significantly associated with gratitude (OR = 1.96, *p* < 0.05), after controlling for solicitation strategies (Model 2). Finally, none of four interaction terms was significant in Model 3. Emotional support still showed a marginally significant association with gratitude (OR = 2.47, *p* = 0.05).

**Table 6.** Logistic regression predicting effects of social support solicitation and provision on gratitude expression.

	Model 1		Model 2		Model 3	
	OR	S.E	OR	S.E	OR	S.E
Emotional support	1.868*	0.274	1.962*	0.279	2.467	0.465
Informational support	1.375	0.214	1.156	0.229	0.948	0.311
Companionship support	0.607	0.433	0.543	0.436	0.539	0.441
Direct support request			1.374	0.262	2.243	0.475
Positive emotional disclosure			0.952	0.232	2.009	0.579
Negative emotional disclosure			1.645	0.265	1.759	0.53
Disclosure with recovery problem			0.933	0.265	0.569	0.429
Direct request* informational support					0.44	0.624
Recovery problem* informational support					0.93	0.568
Pos*Emotional support					2.117	0.493
Neg*Emotional support					0.535	0.56
Intercept	0.045	0.331	0.039	0.331	0.034	0.472

*Note.* *p < 0.05.

## Discussion

Through the behavior of providing particular types of social support, support providers may respond to the way receivers convey their circumstances and needs. These dynamics have not been well studied in an online context, and better understanding them could be helpful in order to guide those using online peer support forums to solicit and provide support more effectively. In a forum for alcohol abuse recovery, we found significant differences in the volume and type of support offered according to recipients’ support solicitation style. Direct solicitations, disclosure of recovery problems, and expression of emotions received the highest volume of supportive replies. Our results also showed that messages that did not reveal recovery problems, did not directly request support, and did not reveal negative emotions were more likely to go without any response from peers. Messages disclosing more mundane thoughts and experiences or that were neutral or positive in valence might have indicated to peers that disclosers were successfully managing their recovery without help.

We also found that the type of support varied according to the solicitation style. As predicted, direct requests for support or descriptions of recovery problems received significantly more informational support than other solicitations, which is consistent with some prior work. This study also showed that emotional support matching was limited to contexts where recipients disclosed positive emotions, with negative emotional disclosures actually receiving more informational support than other disclosure types. Finally, companionship support was most common following direct support requests and disclosures that had mixed negative and positive emotions, which perhaps reflects that support providers felt they needed additional channels of communication in order to provide adequate help. For instance, sharing both positive and negative emotions conveys ambivalence, which may require support providers’ to solicit additional details or clarification. Disambiguation may be more feasible through support exchanges that are more private (i.e. one-to-one messaging), truly synchronous (e.g. face to face; the telephone) or with more nonverbal cues (all nonverbal cues for face to face; vocal cues for the phone).^66,67^

We did not find evidence that matching between solicitation style and support type influenced expression of gratitude. That is, for example, emotional support did not receive more gratitude when it responded to an emotional solicitation, and likewise, informational support did not receive more gratitude when it responded to a direct request for social support. Rather, those who received emotional support responded with more gratitude regardless of their solicitation style.

These findings have implications for clarifying the processes underlying effective support delivery in online recovery groups. Specifically, our findings are potentially consistent with those of Wang and colleagues^53^ in highlighting the relevance of emotional support to building relationships in a recovery context. Their study found that receipt of emotional support increased commitment to an online recovery group. Our findings build on these by showing that receipt of emotional support also manifests between the dyad, as gratitude expression. Prior research has found that gratitude expression can foster mutual obligation and closeness that motivate ongoing interaction and relationship maintenance.^68,69^ Bonding with others is highly beneficial to people who are recovering from AUD, since it compensates for the feelings of isolation that accompany substance dependence.^70^ Taken in combination, our findings of high frequencies of emotional support, and high frequencies of expressed gratitude for such support, are encouraging. In fact, we identified frequencies of emotional support (75% of supportive replies) that exceeded estimates from other studies of online support groups.^71^ This finding could correspond, in part, to the characteristics of the particular social network we examined, where a common bond may have been built by recruitment into a closed social network through a treatment provider, perhaps establishing a relatively high degree of trust and social bonding.

Despite the positive outcomes of emotional support in this and other studies, we found that emotional support was offered unevenly. While it was the most common type of support offered, emotional support was greatly overrepresented after disclosure of positive emotions. This may reflect that emotional support, which includes encouragement, could be used to affirm self-disclosures that express positive affect around recovery progress. The expression of emotional support after positive emotions may also be consistent with emotional contagion, a phenomenon where public emotional expressions produce similar emotional experiences in members of one’s social network.^72-74^

In contrast, negative emotional disclosures received far less emotional support. This finding contrasts with prior work suggesting that displaying vulnerabilities is required to solicit emotional support.^75^ Such findings are potentially problematic. While a range of studies have found health and well-being benefits associated with self-disclosure, including disclosure of negative emotions,^76^ it is not clear if and when these same benefits accrue within a social context that may create an expectation of response. Indeed, receiving support after a disclosure may be especially important for those who disclose negative emotions (relative to those who disclose positive emotions),^59^ which corresponds to findings that affiliation is especially important among those who are distressed.^77^ In the present study, we found that individuals expressed more gratitude for emotional support, and this finding held true after negative emotional disclosures, despite emotional support being offered less frequently in these contexts. It is possible that lay support providers simply misjudge the helpfulness of their support efforts. While recovery problems, direct support requests, and negative emotions all tended to spur a greater number of supportive responses, these same solicitations also all received a lower proportion of emotional support and a higher proportion of informational support than other solicitations.

### Design and training implications

Our findings have practical implications for providing guidance to users of online peer-to-peer support forums. For instance, given the reduced production of emotional support after negative self-disclosures, it may be worthwhile to consider design elements that would spur supporters to provide more compassionate or encouraging responses. For instance, systems may include “light touch” responses that simplify the process of conveying support by allowing for brief supportive responses through pressing a single button.^59,78^ While “like” buttons can have an ambiguous meaning that might make them inappropriate after negative emotional disclosures, other alternatives such as “hugs” may be useful to easily convey social support.^38^ It may also be possible to automate responses based on linguistic characteristics of support solicitations.^79^ For instance, if participants describe negative affect, a message could respond to say, “Negative emotions are a normal part of recovery. We can definitely relate, and we are rooting for you.” For recovery problem messages, a forum moderator could be prompted to review the message and offer a personalized response.

Support delivery is also influenced by the ways that designers and administrators of online communities establish guidance and training for supportive interactions between peers.^8,28^ This guidance may emphasize different ideals of support provision. For instance, some forums train support providers to engage in cognitive reframing, or “debugging” of others’ maladaptive thoughts.^80^ Others guide peers to focus on conveying emotional support, such as through listening to and affirming recipients’ experiences without offering advice.^14,81^ In face-to-face recovery, Alcoholics Anonymous has similar norms of avoiding direct advice.^82^ Training and guidance could enhance emotional support by conveying to participants the value of making support recipients feel their emotions and concerns have been heard and empathetically understood.

Guidance may also address support seekers. For instance, it may be helpful for those seeking support to know that they may receive more feedback from their peers if they disclose their feelings and problems. Communication skills training around introspection and disclosure has been shown to help those in face-to-face mutual support groups for addiction recovery,^83^ and could potentially be incorporated in digital interventions as well.

### Limitations and areas for future research

Several directions for future research emerge from these findings. First, it should be noted that expressing gratitude provides a useful but incomplete window into the utility of receiving social support, and additional outcomes should be considered. Specifically, while recipients may well *appreciate* a form of support in the moment, examining gratitude expression may not indicate which support will hold value over the long term. For instance, receiving advice or cognitive reframing could lack immediate gratification even if it is ultimately useful. It will therefore be helpful to know whether participants who receive more emotional, informational, and companionship support are better able to cope with recovery challenges and less likely to relapse. In addition, as expressing gratitude is important for forming and maintaining a mutually responsive social relationship,^68^ future studies can explore the relationships between supportive message exchanges, gratitude expression, and longer-term outcomes, such as the sustained system usage or group bonding in online support groups.

It is also worth noting that many other factors could contribute to gratitude expression, such as the prior interactions between a dyad, their personal characteristics, and features of their messages that we did not consider here. Finally, much of our social behavior reflects compliance with conventions, including conventions that may call for expressing gratitude as a demonstration of courtesy.^84^ In future study, researchers may wish to consider coding schemes that can distinguish expressions of gratitude that reflect courtesy from those that demonstrate genuine appreciation. For instance, courtesy might be indicated by short, simple replies, whereas appreciation might be indicated through more detailed and thoughtful replies, especially those that respond to the content of the received support or describe how the receiver intends to act upon that support in the future. Additional methods including in-depth interviewing may also play a role in better understanding the motivations behind gratitude expression.

There are also a number of additional dimensions of solicitations and social support that we did not distinguish in our coding but that may be relevant to understanding dynamics of support exchange. For instance, we considered emotion along a single dimension of valence, but some research shows that specific negative emotions such as anger, sadness, and anxiety may call for different support responses.^24^ Legitimating feelings of sadness may be effective, whereas challenging or prompting elaboration about unwarranted anger may be more helpful.^85^ In addition, certain types of support may be appropriate in light of the controllability of the recipient’s situation. Situations in which individuals grapple with circumstances out of their control, such as the death of a loved one, may call for emotional support, but individuals may prefer informational resources in order to address more controllable issues, like treatment decisions.^86^ There may also be individual-level traits that moderate the reaction to received support, such as self-esteem.^78^

In addition, support reception may be influenced by some qualities of support that we did not consider. Some work has shown that support may effectively boost morale and foster coping when it takes a non-directive approach that affirms the recipients’ autonomy. In contrast, support that attempts to control the recipients’ coping strategy, such as through prescriptive advice (e.g. “You should …”), can have negative consequences.^87,88^ Informational support can also have high value when it reflects the first-hand experience of peers who have faced similar challenges.^89^ Future work should compare receptivity to informational support offered as a suggestion versus as a directive, and that calls on individual experience or does not.

Last but not least, it is unclear whether norms for soliciting and providing social support have been established within the online support group itself. Social dynamics within the group may influence decisions about what types of support to give or how to solicit it.^32^ Through regular posting, high-profile members may model social support-related behavior for new members.^90^ In a similar way, replies from moderators, who serve as experts in the group, may also play a role in establishing group norms.

## Conclusions

As digital support forums expand their reach, it is critical to understand how individuals needing support can best obtain it. Unlike those attending face-to-face support groups, individuals rely heavily on text to express their needs to peers in online support groups. This study suggests the volume and types of social support vary depending on text-based solicitation strategies. Consistent with the “triage” model, support often goes to individuals who explicitly express they are most in need, including individuals who disclose negative emotions or recovery problems, or directly ask for help. In contrast, evidence of “matching” was inconsistent. There was more informational support only after direct requests and disclosure of recovery problems, which may be consistent with matching. However, emotional support was only matched to positive emotional disclosures, not negative ones. Finally, this study also indicates that emotional support plays an important role in spurring participants’ expression of gratitude toward their support providers. These findings deepen our understanding about the social support process in online AUD forums, and have implications for online support group design and monitoring.

## References

[1] CDC. Alcohol use and health, http://www.cdc.gov/alcohol/fact-sheets/alcohol-use.htm (2016, accessed 25 Feb 2016).

[2] Rondó J and Feliz J. Survey: Ten percent of American adults report being in recovery from substance abuse or addiction, https://www.oasas.ny.gov/pio/press/20120306Recovery.cfm (2012, accessed 20 March 2016).

[3] Abuse S. Mental Health Services Administration (SAMHSA). *Results from the 2008 National Survey on Drug Use and Health: National Findings. Office of Applied Studies. NSDUH Series H-36, HHS Publication No. SMA 09–4434*. Rockville, MD: SAMHSA, 2009.

[4] Dobkin PL Civita MD Paraherakis A The role of functional social support in treatment retention and outcomes among outpatient adult substance abusers. Addiction 2002; 97: 347–356. 11964111 10.1046/j.1360-0443.2002.00083.x

[5] Pauley PM Hesse C The effects of social support, depression, and stress on drinking behaviors in a college student sample. Commun Stud 2009; 60: 493–508.

[6] Schomerus G Lucht M Holzinger A The stigma of alcohol dependence compared with other mental disorders: A review of population studies. Alcohol Alcohol 2011; 46: 105–112. 21169612 10.1093/alcalc/agq089

[7] Grant DS and Dill-Shackleford KE. Using social media for sobriety recovery? Preferences, beliefs, behaviors, and surprises from users of online and social media sobriety support. *Psychol Pop Media Cult* (In press).

[8] Fisher EB Ballesteros J Bhushan N Key features of peer support in chronic disease prevention and management. Health Affairs 2015; 34: 1523–1530. 26355054 10.1377/hlthaff.2015.0365

[9] Heisler M Vijan S Makki F Diabetes control with reciprocal peer support versus nurse care management: A randomized trial. Ann Intern Med 2010; 153: 507–515. 20956707 10.7326/0003-4819-153-8-201010190-00007 PMC4117390

[10] Namkoong K McLaughlin B Yoo W The effects of expression: How providing emotional support online improves cancer patients’ coping strategies. J Natl Cancer Inst Monogr 2013; 47: 169–174. 10.1093/jncimonographs/lgt033 PMC3881999 24395987

[11] Wang Y-C Kraut RE Levine JM Eliciting and receiving online support: Using computer-aided content analysis to examine the dynamics of online social support. J Med Internet Res 2015; 17. 10.2196/jmir.3558 PMC4419194 25896033

[12] Wright KB. Communication in health-related online social support groups/communities: A review of research on predictors of participation, applications of social support theory, and health outcomes. *Rev Communic Res* 2016; 4: 65–87.

[13] Fox S. 2012 Health Survey Data, http://www.pewinternet.org/2013/02/11/2012-health-survey-data/ (2013, accessed 20 March 2016).

[14] Baumel A Schueller SM Adjusting an available online peer support platform in a program to supplement the treatment of perinatal depression and anxiety. JMIR Ment Health 2016; 3. 10.2196/mental.5335 PMC4820657 27001373

[15] Lane JD Wegner DM The cognitive consequences of secrecy. J Pers Soc Psychol 1995; 69: 237.

[16] Shaw BR McTavish F Hawkins R Experiences of women with breast cancer: Exchanging social support over the CHESS computer network. J Health Commun 2000; 5: 135–159. 11010346 10.1080/108107300406866

[17] DeAndrea D Anthony J Online peer support for mental health problems in the United States: 2004–2010. Psychol Med 2013; 43: 2277–2288. 23410539 10.1017/S0033291713000172 PMC4327823

[18] Joinson AN Self-esteem, interpersonal risk, and preference for e-mail to face-to-face communication. Cyberpsychol Behav 2004; 7: 472–478. 15331035 10.1089/cpb.2004.7.472

[19] Walther JB Computer-mediated communication impersonal, interpersonal, and hyperpersonal interaction. Commun Res 1996; 23: 3–43.

[20] Walther JB Pingree S Hawkins RP Attributes of interactive online health information systems. J Med Internet Res 2005; 7: e33. 15998624 10.2196/jmir.7.3.e33 PMC1550659

[21] Tanis M Health-related on-line forums: What's the big attraction? J Health Commun 2008; 13: 698–714. 18958781 10.1080/10810730802415316

[22] Rains SA Peterson EB Wright KB Communicating social support in computer-mediated contexts: A meta-analytic review of content analyses examining support messages shared online among individuals coping with illness. Commun Monogr 2015; 82: 403–430.

[23] Mo PK Coulson NS Developing a model for online support group use, empowering processes and psychosocial outcomes for individuals living with HIV/AIDS. Psychol Health 2012; 27: 445–459. 21854088 10.1080/08870446.2011.592981

[24] Lieberman MA Goldstein BA Not all negative emotions are equal: The role of emotional expression in online support groups for women with breast cancer. Psychooncology 2006; 15: 160–168. 15880627 10.1002/pon.932

[25] Gustafson DH Hawkins R Pingree S Effect of computer support on younger women with breast cancer. J Gen Intern Med 2001; 16: 435–445. 11520380 10.1046/j.1525-1497.2001.016007435.x PMC1495237

[26] Allen C Vassilev I Kennedy A Long-term condition self-management support in online communities: A meta-synthesis of qualitative papers. J Med Internet Res 2016; 18. 10.2196/jmir.5260 PMC4807245 26965990

[27] Lepore SJ Glaser DB Roberts KJ On the positive relation between received social support and negative affect: A test of the triage and self-esteem threat models in women with breast cancer. Psychooncology 2008; 17: 1210–1215. 18613318 10.1002/pon.1347

[28] Lloyd-Evans B Mayo-Wilson E Harrison B A systematic review and meta-analysis of randomised controlled trials of peer support for people with severe mental illness. BMC Psychiatry 2014; 14: 1. 10.1186/1471-244X-14-39 PMC3933205 24528545

[29] Mohr DC Burns MN Schueller SM Behavioral intervention technologies: Evidence review and recommendations for future research in mental health. Gen Hosp Psychiatry 2013; 35: 332–338. 23664503 10.1016/j.genhosppsych.2013.03.008 PMC3719158

[30] Suler J The online disinhibition effect. Cyberpsychol Behav 2004; 7: 321–326. 15257832 10.1089/1094931041291295

[31] Jiang L Bazarova NN Hancock JT The disclosure–intimacy link in computer-mediated communication: An attributional extension of the hyperpersonal model. Human Commun Res 2011; 37: 58–77.

[32] Smithson J Sharkey S Hewis E Problem presentation and responses on an online forum for young people who self-harm. Discourse Stud 2011; 13: 487–501.

[33] Morrow PR Telling about problems and giving advice in an Internet discussion forum: Some discourse features. Discourse Stud 2006; 8: 531–548.

[34] Lewallen AC Owen JE Bantum EOC How language affects peer responsiveness in an online cancer support group: Implications for treatment design and facilitation. Psychooncology 2014; 23: 766–772. 24519856 10.1002/pon.3477 PMC4082444

[35] Cutrona CE and Russell DW. Type of social support and specific stress: Toward a theory of optimal matching. In: Sarason BR, Sarason IG, and Pierce GR (eds) *Social support: An interactional view*, 1990, pp. 319–366. Oxford, UK: John Wiley.

[36] Jourard SM Lasakow P Some factors in self-disclosure. J Abnorm Soc Psychol 1958; 56: 91. 10.1037/h0043357 13501977

[37] Burke M and Develin M. Once more with feeling: Supportive responses to social sharing on Facebook. In: *Proceedings of the 19th ACM Conference on Computer-Supported Cooperative Work & Social Computing 2016*, ACM, pp.1462-1474.

[38] Ahmadi M, Schneider ME, Kadam R, et al. Designing paralinguistic digital affordances for social support. In: *Proceedings of the 19th ACM Conference on Computer Supported Cooperative Work and Social Computing Companion 2016*, ACM, pp.221-224.

[39] Horowitz LM Krasnoperova EN Tatar DG The way to console may depend on the goal: Experimental studies of social support. J Exp Soc Psychol 2001; 37: 49–61.

[40] Marigold DC Cavallo JV Holmes JG You can’t always give what you want: The challenge of providing social support to low self-esteem individuals. J Pers Soc Psychol 2014; 107: 56. 24956314 10.1037/a0036554

[41] Priem JS Solomon DH Emotional support and physiological stress recovery: The role of support matching, adequacy, and invisibility. Commun Monogr 2015; 82: 88–112.

[42] Rimé B. Interpersonal emotion regulation. In: *Handbook of emotion regulation*. New York: Guilford Press, 2007, pp.466-468.

[43] Cutrona CE Shaffer PA Wesner KA Optimally matching support and perceived spousal sensitivity. J Fam Psychol 2007; 21: 754. 18179347 10.1037/0893-3200.21.4.754

[44] Vlahovic TA, Wang Y-C, Kraut RE, et al. Support matching and satisfaction in an online breast cancer support community. In: *Proceedings of the 32nd annual ACM conference on Human factors in computing systems 2014*, ACM, pp.1625-1634.

[45] Batenburg A Das E An experimental study on the effectiveness of disclosing stressful life events and support messages: When cognitive reappraisal support decreases emotional distress, and emotional support is like saying nothing at all. Plos One 2014; 9: e114169. 25531509 10.1371/journal.pone.0114169 PMC4273978

[46] Burleson BR. Explaining recipient responses to supportive messages. *New Dir Interpers Commun Res* 2010; 159–179.

[47] Uchino BN. Social support and physical health: Understanding the health consequences of relationships, Yale: Yale University Press, 2004.

[48] Kowitt SD Urlaub D Guzman-Corrales L Emotional support for diabetes management: An international cross-cultural study. Diabetes Educ 2015; 41: 291–300. 25722064 10.1177/0145721715574729

[49] Chuang KY and Yang CC. Helping you to help me: exploring supportive interaction in online health community. In: *Proceedings of the American Society for Information Science and Technology*, 1 November 2010, Vol. 47, pp. 1–10.

[50] Klaw E Dearmin Huebsch P Humphreys K Communication patterns in an on-line mutual help group for problem drinkers. J Commun Psychol 2000; 28: 535–546.

[51] MacLean D, Gupta S, Lembke A, et al. Forum77: An analysis of an online health forum dedicated to addiction recovery. In: *Proceedings of the 18th ACM Conference on Computer Supported Cooperative Work & Social Computing 2015*, ACM, pp.1511–1526.

[52] Coulson NS Sharing, supporting and sobriety: A qualitative analysis of messages posted to alcohol-related online discussion forums in the United Kingdom. J Subst Use 2014; 19: 176–180.

[53] Wang Y-C, Kraut R and Levine JM. To stay or leave?: The relationship of emotional and informational support to commitment in online health support groups. In: *Proceedings of the ACM 2012 conference on Computer Supported Cooperative Work 2012*, ACM, pp.833–842.

[54] Wood AM Froh JJ Geraghty AW Gratitude and well-being: A review and theoretical integration. Clin Psychol Rev 2010; 30: 890–905. 20451313 10.1016/j.cpr.2010.03.005

[55] Dickens L DeSteno D The grateful are patient: Heightened daily gratitude is associated with attenuated temporal discounting. Emotion 2016; 16: 421. 27018609 10.1037/emo0000176

[56] Chen G Does gratitude promote recovery from substance misuse? Addict Res Theory 2016, pp. 1–8.27429607

[57] Ghandeharioun A Azaria A Taylor S “Kind and Grateful”: A context-sensitive smartphone app utilizing inspirational content to promote gratitude. Psychol Wellbeing 2016; 6: 1–21. 10.1186/s13612-016-0046-2 PMC4932143 27441170

[58] Bazarova NN Public intimacy: Disclosure interpretation and social judgments on Facebook. J Commun 2012; 62: 815–832.

[59] Bazarova NN, Choi YH, Schwanda Sosik V, et al. Social sharing of emotions on Facebook: Channel differences, satisfaction, and replies. In: *Proceedings of the 18th ACM Conference on Computer Supported Cooperative Work & Social Computing 2015*, ACM, pp.154–164.

[60] Forest AL Wood JV When social networking is not working individuals with low self-esteem recognize but do not reap the benefits of self-disclosure on Facebook. Psychol Sci 2012 0956797611429709. 10.1177/0956797611429709 22318997

[61] Wiseman RL Schenck-Hamlin W A multidimensional scaling validation of an inductively-derived set of compliance-gaining strategies. Commun Monogr 1981; 48: 251–270.

[62] Cohen S and Wills TA. Stress, social support, and the buffering hypothesis. *Psychological Bulletin* 1985; 98: 310. 3901065

[63] Bambina A. *Online social support: The interplay of social networks and computer-mediated communication*. Cambria Press, 2007.

[64] Jacobson DE. Types and timing of social support. *J Health Soc Behav* 1986; 250–264. 3772062

[65] Gustafson DH McTavish FM Chih M-Y A smartphone application to support recovery from alcoholism: A randomized clinical trial. JAMA Psychiatry 2014; 71: 566–572. 24671165 10.1001/jamapsychiatry.2013.4642 PMC4016167

[66] Tidwell LC Walther JB Computer-mediated communication effects on disclosure, impressions, and interpersonal evaluations: Getting to know one another a bit at a time. Human Commun Res 2002; 28: 317–348.

[67] Walther JB and Parks MR. Cues filtered out, cues filtered. In: *Handbook of Interpersonal Communication*, 2002, Vol. 3, pp.529–563.

[68] Algoe SB Find, remind, and bind: The functions of gratitude in everyday relationships. Soc Pers Psychol Compass 2012; 6: 455–469.

[69] Algoe SB Haidt J Gable SL Beyond reciprocity: Gratitude and relationships in everyday life. Emotion 2008; 8: 425. 18540759 10.1037/1528-3542.8.3.425 PMC2692821

[70] Inagaki TK Eisenberger NI Giving support to others reduces sympathetic nervous system-related responses to stress. Psychophysiology 2016; 53: 427–435. 26575283 10.1111/psyp.12578

[71] Keating DM Spirituality and support: A descriptive analysis of online social support for depression. J Relig Health 2013; 52: 1014–1028. 22322336 10.1007/s10943-012-9577-x

[72] Fowler JH Christakis NA Dynamic spread of happiness in a large social network: Longitudinal analysis over 20 years in the Framingham Heart Study. BMJ 2008; 337: a2338. 19056788 10.1136/bmj.a2338 PMC2600606

[73] Gable SL Reis HT Impett EA What do you do when things go right? The intrapersonal and interpersonal benefits of sharing positive events. J Pers Soc Psychol 2004; 87: 228. 15301629 10.1037/0022-3514.87.2.228

[74] Hatfield E Cacioppo JT Rapson RL Emotional contagion, Cambridge: Cambridge University Press, 1994.

[75] Newman MW, Lauterbach D, Munson SA, et al. It's not that i don't have problems, i'm just not putting them on facebook: Challenges and opportunities in using online social networks for health. In: *Proceedings of the ACM 2011 conference on Computer supported cooperative work 2011*, ACM, pp.341-350.

[76] Pennebaker JW Chung CK Expressive writing: Connections to physical and mental health. *In:* Oxford Handbook of Health Psychology, Oxford: Oxford University Press, 2011, pp. 417–437.

[77] Cousin G Mast MS Jaunin-Stalder N Finding the right interactional temperature: Do colder patients need more warmth in physician communication style? Soc Sci Med 2013; 98: 18–23. 24331877 10.1016/j.socscimed.2013.08.034

[78] Wohn DY Carr CT Hayes RA How affective is a “Like”?: The effect of paralinguistic digital affordances on perceived social support. Cyberpsychol Behav Soc Network 2016; 19: 562–566. 10.1089/cyber.2016.0162 27635443

[79] Shibata T, Egashira Y and Kurohashi S. Chat-like conversational system based on selection of reply generating module with reinforcement learning. In: *Situated Dialog in Speech-Based Human-Computer Interaction*. Springer, 2016, pp.63-69.

[80] Morris RR Schueller SM Picard RW Efficacy of a web-based, crowdsourced peer-to-peer cognitive reappraisal platform for depression: Randomized controlled trial. J Med Internet Res 2015; 17. 10.2196/jmir.4167 PMC4395771 25835472

[81] Lepore SJ Buzaglo JS Lieberman MA Comparing standard versus prosocial internet support groups for patients with breast cancer: A randomized controlled trial of the helper therapy principle. J Clin Oncol 2014; 32: 4081–4086. 25403218 10.1200/JCO.2014.57.0093 PMC4265118

[82] Borkman T Sharing experience, conveying hope: Egalitarian relations as the essential method of Alcoholics Anonymous. Nonprofit Manage Leadership 2006; 17: 145–161.

[83] Anderson JG Gilbert FS Communication skills training with alcoholics for improving performance of two of the Alcoholics Anonymous recovery steps. J Stud Alcohol 1989; 50: 361–367. 2755136 10.15288/jsa.1989.50.361

[84] Brown P Levinson SC Politeness: Some universals in language usage, Cambridge: Cambridge University Press, 1987.

[85] Burleson BR and Goldsmith DJ. How the Comforting Process I/Vorles: Alleviating Emotional Distress through Conversationally Induced Reappraisals. In: *Handbook of communication and emotion: Research, theory, applications, and contexts.* 1997, pp.245–280.

[86] Cutrona CE Stress and social support—In search of optimal matching. J Soc Clin Psychol 1990; 9: 3–14.

[87] Harber KD Schneider JK Everard KM Directive support, nondirective support, and morale. J Soc Clin Psychol 2005; 24: 691.

[88] Stewart DW Gabriele JM Fisher EB Directive support, nondirective support, and health behaviors in a community sample. J Behav Med 2012; 35: 492–499. 21877174 10.1007/s10865-011-9377-x

[89] Perry BL Pescosolido BA Social network activation: The role of health discussion partners in recovery from mental illness. Soc Sci Med 2015; 125: 116–128. 24525260 10.1016/j.socscimed.2013.12.033 PMC4110193

[90] Pedersen S Smithson J Membership and activity in an online parenting community. Handbook of research on discourse behavior and digital communication: Language structures and social interaction, Hershey, PA: IGI Global, 31 May 2010, pp. 88–103.

